# Capsaicin: An Uncommon Exposure and Unusual Treatment

**DOI:** 10.5811/cpcem.2019.3.41231

**Published:** 2019-05-20

**Authors:** Onur M. Yenigun, Mark Thanassi

**Affiliations:** * Stanford University Medical Center, Department of Emergency Medicine, Palo Alto, California; † Kaiser Permanente Medical Center, Department of Emergency Medicine, Santa Clara, California

## Abstract

Capsaicin, the active component of chili peppers, is an alkaloid that causes tissue irritation and burning especially upon contact with mucous membranes. While favored in certain cuisines around the world, it has also been weaponized in the form of pepper sprays and bear repellents. When significant capsaicin exposures occur, patients may present to the emergency department; thus, providers should be prepared to manage these cases effectively. In this case report we discuss an unusual exposure of capsaicin to the vaginal mucosa with successful treatment.

## INTRODUCTION

In 2017 the American Association of Poison Control Centers reported 2,229 exposures to capsicum-containing peppers, 215 of which were treated in a healthcare facility. An additional 3,320 exposures to capsicum defense sprays were reported, and 833 of these sought medical care.^1^ Pepper sprays are prevalent and commercially available in the United States. They contain the active ingredient oleoresin capsicum, which is an oily extract composed primarily of capsaicinoids. These hydrophobic, fat-soluble phenols are produced by chili peppers and elicit significant pain and burning upon tissue and mucous-membrane exposure.^2^ Capsaicinoids act as a direct irritant, in addition to causing secondary neurogenic inflammation elicited by capsaicin’s (Figure) interaction with sensory neurons via the vanillin receptor subtype 1 (TRPV).^3^ The hydrophobic nature of these compounds explains why water and other hydrophilic solvents are minimally effective in alleviating their noxious effects.^4^

While oral mucosal exposures to capsaicin are fairly common, other body areas can be affected in situations such as military training, police actions, and self-defense where non-lethal means of subduing individuals is required. Effects vary based on the anatomic site involved. Dermatological exposures induce burning, hyperalgesia and erythema. Ophthalmic effects include blepharospasm, conjunctivitis, corneal erosions, and periorbital edema, whereas inhalation often results in dyspnea and a burning sensation in the chest.^5^,^6^

Various strategies for neutralizing the pain and burning of capsaicin exposure have been explored including baby shampoo, mixtures of detergent solutions, milk, topical anesthetics, and antacid solutions such as calcium carbonate.^7^ Specialized decontaminants such as Sudecon wipes have been developed, as well as SABRE Decon and BioShield sprays commonly used by law enforcement. These methods use detergents, surfactants, or other hydrophobic molecules with the ability to displace and neutralize the compound. The following case report describes an unusual capsaicin exposure and subsequent effective treatment in the emergency department (ED).

## CASE REPORT

A 22-year-old female presented to the ED with the chief complaint of severe vaginal pain and burning. The patient reported that she was a security guard who routinely carried pepper spray in her bag. She was not aware that the canister had discharged into the bag containing personal items including tampons. When the patient subsequently inserted the tampon she immediately experienced severe vaginal mucosal burning that was not relieved after removal of the tampon; so she sought medical care. The time of exposure to ED arrival was approximately 20 minutes.

Initial presentation revealed a patient in significant distress, crying out in pain. Vital signs were notable for mild tachycardia and otherwise within normal limits. Pertinent exam findings included labial and vaginal erythema and extreme sensitivity. The pepper spray canister was analyzed and found to contain capsaicin without other significant irritant or corrosive additives.

Analgesia was attempted with four milligrams (mg) of intravenous (IV) morphine, which had no observed effect over a 15-minute period. Management was then directed towards neutralizing and displacing the capsaicin from the mucosa. A medium-sized, disposable plastic speculum was obtained and lubricated with 2% lidocaine jelly while two tampons were presoaked in cold, pasteurized 2% skim milk. After patient consent, a standard speculum exam was performed, which was notable for vaginal mucosal erythema without ulcerations or bleeding. The saturated tampons were then placed in-line within the vaginal canal and the speculum was removed leaving the tampons in place with the strings externalized to permit easy removal. An icepack and milk-soaked towel were then placed over the groin.

The patient rapidly achieved significant pain relief within 5–7 minutes with a reported reduction in her pain scale from 10/10 to 3/10. After an approximately 15-minute dwell time the tampons were removed; the patient was observed for a brief period and then discharged to home in stable condition.

## DISCUSSION

Pain management in the ED commonly involves IV and oral medications. This case, however, highlights the fact that topical irritants can be more effectively managed via direct anesthesia and displacement of the offending compound. Topical anesthetics such as lidocaine alleviate pain through inhibition of sensory neurons via sodium channel blockade. Viscous preparations more effectively adhere to the involved tissue, but will be absorbed by mucosal tissue so it is important to keep in mind the toxic dose based on patient weight. Furthermore, lidocaine uptake will be increased in abraded tissue and should be kept in mind. Toxicity can occur at doses over 5–7 mg per kilogram and may result in cardiac arrhythmias or seizures.

**CPC-EM Capsule**

What do we already know about this clinical entity?

Capsaicin is a hydrophobic, fat-soluble compound that acts as a direct irritant to mucosal surfaces, causing significant pain and burning upon exposure.

What makes this presentation of disease reportable?

This is an unusual exposure of capsaicin to the vaginal mucosa with a unique and effective means of management.

What is the major learning point?

Capsaicinoids are best displaced by hydrophobic solutes. Furthermore, topical anesthetics such as lidocaine are effective in temporarily alleviating pain via inhibition of sensory neurons.

How might this improve emergency medicine practice?

This report adds to the emergency physician’s toolbox by providing a quick and effective means of managing an exposure to capsaicin with frequently available products.

Strategies to directly displace an irritant must take into account the nature of the compound. Hydrophilic substances such as water will have little to no efficacy in displacing hydrophobic molecules such as capsaicin from sensory receptors. Milk, on the other hand, is readily available in the hospital setting and contains the hydrophobic protein casein, which effectively displaces capsaicin, quickly relieving pain. Ten percent glucose solutions, also readily available in the ED, have additionally been shown to decrease the pain associated with capsaicin exposure; however, this has only been studied in the oral cavity and may not be generalizable to other areas of the body.^8^

As with all patient chemical exposures, measures should be taken to limit healthcare worker contamination. Appropriate personal protective equipment such as gloves, gowns, and face shields should be used.

## CONCLUSION

While capsaicin exposure is not uncommon and typically involves the face, this case illustrates a novel approach to managing an unusual case of vaginal mucosal involvement and presents effective strategies for pain management using readily available therapies. As emergency physicians we must be prepared to quickly adapt to challenging cases and think outside the box. The ideal or most accessible treatment may not always be in your department’s medical cabinet.

## Figures and Tables

**Figure:**
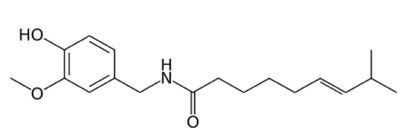
Capsaicin molecular structure, C_18_H_27_NO_3_. *C,* carbon, *H;* hydrogen; *N,* nitrogen; *O,* oxygen.
